# Validation of an instrument for measuring satisfaction of patients undergoing hemodialysis

**DOI:** 10.1186/s12913-017-2251-y

**Published:** 2017-05-03

**Authors:** Mauricio Sanabria-Arenas, Julia Tobón- Marín, María Claudia Certuche-Quintana, Ricardo Sánchez-Pedraza

**Affiliations:** 1 Renal Therapy Services (RTS), Bogotá D.C, Colombia; 2 Laboratorios Baxter, Bogotá D.C, Colombia; 3 School of Medicine. Universidad Nacional de Colombia, Bogotá D.C, Colombia; 4 Edificio City Business, Transversal 23 # 97-73, 6° Piso, Bogotá, Colombia

**Keywords:** Patient satisfaction, Questionnaires, Hemodialysis units, Chronic kidney failure

## Abstract

**Background:**

Patients’ satisfaction is an indicator of the quality of healthcare services. Its measurement involves developing and validating complex instruments. The purpose of this study was to validate a scale for measuring hemodialysis patients’ satisfaction with the provided care, the Scale for Evaluation of Hemodialysis Patient’s Satisfaction with Service provided at a Chronic Kidney Disease Unit (or ESUR-HD, its acronym in Spanish).

**Methods:**

The instrument was applied to 370 patients undergoing hemodialysis for undertaking exploratory and confirmatory analyses, internal consistency assessment, and Rasch analysis. In order to assure test-retest reliability, the instrument was applied once again to 54 patients after 2 days. Convergent validity was assessed by estimating correlation coefficients based on the results of 2 instruments (ESUR-HD and SDIALOR) simultaneously applied in 70 patients. Sensitivity to change was assessed in 40 patients by comparing the scale scores before and after an intervention consisting of improved care conditions.

**Results:**

In the 44 items of the scale a 9-factor structure was found (1: Facilities and organization of the service. 2: Care provided by the attending nurses and/or nursing assistants. 3: Attention to psychological and administrative issues. 4: Contact and social work personnel. 5: Medical attention and care. 6: Nutritional attention and care. 7: Medications supply and quality. 8: Features of the admission process. 9: Attention and care provided by head nurses). Chronbach alpha for the scale was 0.96. Lin’s concordance correlation for the whole scale was 0.85. Although statistically different from 0, low correlation values with dimensions from another scale measuring the same attribute were found. The scale could detect construct changes through increased scores in specific dimensions following an intervention aimed at enhancing satisfaction. Rasch analysis located improperly fit items and suggested reducing items measurement levels. Despite the effect encountered, Rasch analysis showed the scale might not capture variability in upper attribute levels.

**Conclusion:**

The ESUR-HD scale measures hemodialysis patients’ satisfaction in one dimension with 9 domains. Validity and reliability are adequate. The instrument may detect changes in the construct. Subsequent versions of the scale should include new items allowing improved discrimination amongst high satisfaction levels.

**Trial registration:**

10.1186/ISRCTN45318400. April 05, 2017

## Background

Satisfaction with a healthcare service has been defined as the quality of an offered service as perceived by the patient, and is a performance indicator of healthcare organizations [1]. Such satisfaction is a top consideration when measuring healthcare and services to fulfill patients’ expectations and values [2]. It has been proposed that any quality evaluation of healthcare services include a patient’s satisfaction, instead of being restricted to conventional indicators such as morbidity and mortality [3]. Satisfaction is a complex concept which depends on an individual patient’s characteristics (*e.g.*, lifestyle, previous healthcare experiences, values), as well as on social characteristics, particular disease issues, and healthcare services (follow-up, treatment adherence, health services stability) [4].

Healthcare quality is an increasingly important issue in medicine [5–8], especially regarding chronic conditions, as is the case of end-stage kidney disease. It has been seen that a patient’s satisfaction is associated with adherence to therapy (*i.e.*, increased satisfaction leads to improved adherence) [9].

Concerning kidney disease, quality improvement regards not just dialysis therapy but also related products and services [10]. Amongst such services, those associated with psychosocial issues are particularly important, as it has been shown that outcomes such as mortality are associated with depression, lack of psychosocial support, and patients’ perceptions about their disease [11–13]. Patient satisfaction with chronic kidney care and caregivers, it has been said, also relates to quality of life as perceived by the patient [9, 11, 14].

Another remarkable aspect of a disease of this kind is that as a result of the long-term and technical peculiarities of the therapy, the patients and the treating team build relationships that are usually close and lasting [13]. It was not long ago that above-mentioned peculiarities of dialysis therapy were included in the tools for measuring patient’s satisfaction with care provided [15–17].

Some research into peritoneal dialysis services have shown that a patient’s satisfaction is associated to the depth of information offered by the treating team, the compassion with which the service is provided, how efficient the dialysis elements supply is, and the presence of a nurse [16]. It has also been described that patients undergoing peritoneal dialysis show higher degrees of satisfaction than those undergoing hemodialysis and that their satisfaction could be improved by offering them information about potential adverse therapy events [18] and about peritoneal dialysis as an option [19]. A study reported that a negative perception of the treating nephrologist is associated with poor therapy adherence [15].

Despite the relevance of the subject, not many validated instruments are available for evaluating satisfaction among kidney disease patients undergoing dialysis therapy. The *Choices* for Healthy Outcomes in Caring for End-Stage Renal Disease (CHOICE) [17] is an instrument that has been used for comparing satisfaction with the type of dialysis therapy [19], and as the basis for the development of other instruments. Other instruments for evaluating satisfaction in patients undergoing dialysis are the Satisfaction of Patients in Chronic Dialysis (SEQUS) [18], the SDIALOR (*Satisfaction des patients dialysés en Lorraine*) [1], the Client Satisfaction Questionnaire (CSQ) [20], the Customer Quality Index (CQ-index), the Renal Treatment Satisfaction Questionnaire (RTSQ) [21] and the Consumer Assessment of Healthcare Providers and Systems In-Center Hemodialysis (CAHPS-ICH) survey [22].

Based on what has been stated so far, it may be argued that measuring a patient’s satisfaction: [1] is an essential element for evaluating the quality of healthcare services; [2] may be used as an institutional performance indicator; and [3] is related to a patient’s quality of life and adherence to therapy. Until recently, it has not been possible to measure this construct in Colombia by means of instruments with known psychometric properties. Thus, validating a questionnaire allowing the assessment of hemodialysis patients’ satisfaction in a valid and reliable way in Colombia is deemed important and is the objective of this study.

## Methods

The 44 items scale for evaluating satisfaction with the service offered at a hemodialysis unit to chronic kidney disease patients (ESUR-HD) was initially developed by a group of nephrologists, nurses, and patients. Following a review of the literature, potential variables or dimensions associated with chronic kidney patients’ satisfaction were identified.

Four focus groups - each with 3 nurses, a nephrologist and two persons of the administrative area - were done in four different regions of the country. They asked what were the main aspects that could influence a patient’s satisfaction. The backbone of the evaluation were the processes and procedures of clinical care in hemodialysis of Renal Therapy Services.

Through the focus groups, the following dimensions were defined: overall satisfaction with the services (3 items), personnel at the unit (24 items), medications and supplies (4 items), facilities and processes (13 items), and phone contact (6 items). The instrument was designed as a phone survey. Following a preliminary trial, its initial structure was modified by removing 6 items because of redundancy or poor relevance. This has been the only available version of the scale and is the one used in this validation. Answer options for each of the 44 items are rated 1 to 5 by means of a Likert scale ranging from “Very unsatisfied” to “Very satisfied.” A final score is obtained by non-weighted sum of the score given to each item; accordingly and as a result of items structure, higher scores reflect increased patient satisfaction. The time it takes to complete the instrument is 15 min (median time).

The instrument was applied to a sample of patients (*n* = 370) undergoing therapy at a hemodialysis program during 2013; each patient was asking by telephone about their willingness to answer the survey and 6% of them refused to answer. Such sample was used to perform an exploratory factorial analysis, a structural-equation confirmatory analysis, internal consistency, and Rasch analysis. To evaluate convergent validity, the SDIALOR scale [1] was simultaneously applied in a subgroup of patients (*n* = 70) of the initial sample; this scale consists of 7 domains (organization of medical care, relationship between nephrologists and general practitioner, locational characteristics, accessibility, care provided by the health personnel, information provided by the doctor, problem solving, overall satisfaction) and shows levels of internal consistency above 0.7 in different domains. It was used because it is the only cross-culturally adapted instrument to measure patient satisfaction with available care for renal disease in Colombia [23]. Test-retest reliability was also evaluated by applying again the ESUR-HD scale 2 days after the initial assessment in a subgroup (*n* = 54) of the 370 patients. This time period was used considering the scale length and the recommendation of some authors on applications to assess test-retest reliability [24]. In order to establish the sensitivity to change, the scale was applied to 40 patients before and after an intervention. In other words, the patients were evaluated to measure their satisfaction in a hemodialysis center, and then were re-evaluated one (1) month after being transferred to a new renal clinic within a hospital - with remodeled spaces, waiting rooms, hemodialysis equipment with newer technology along with notably better prepared healthcare personnel more familiar, expert and dedicated to the patient’s care.

The data from the study and the full instrument may be required from the principal investigator Mauricio Sanabria: mauricio_sanabria@baxter.com.

### Statistical analyses

Considering that the latent dimensions structure of the instrument was purely theoretical, an exploratory factorial analysis was carried out, taking into account the ordinal nature of the variables (each item being rated on a Likert-type scale), using a minimal residues factorization method on a polychoric correlation matrix. The parallel analysis method [22, 25] was applied for determining the number of factors. An orthogonal rotation (Varimax) was used to improve factors interpretability. Structural equations from polychoric correlation matrices and asymptotic covariance matrices were used for the confirmatory factorial analysis (which was done considering the ordinal nature of the items’ qualification). As an estimation method, diagonally weighted least squares were used, assuming no normal data distribution. Data fit was assessed for 2 model types: one guided by the exploratory factorial analysis and one suggested by the changes in modification indexes. Criteria for considering whether the models fit was adequate were as follows [23, 26]: Ratio of *Χ* ^2^ to degrees of freedom ($$ \raisebox{1ex}{${X}^2$}\!\left/ \!\raisebox{-1ex}{$ df$}\right. $$) < 3, Tucker-Lewis index (TLI) and comparative fit index (CFI) > 0.9, and root mean square error of approximation (RMSEA) < 0.8. In addition, both Bayesian information criteria (BIC) and Akaike information criteria (AIC) were calculated, lower values suggesting better model fit. For estimating the sample size for the factorial analyses with this type of covariance structures, the recommendation of having at least 250 observations was taken into account [24, 27].

To evaluate the internal consistency of the scales, factors, and items, Cronbach alpha was calculated for the whole scale as well as for each domain suggested by the factorial analysis and for the scale deleting each of the items. A sample of 257 subjects, each answering 44 items, would allow 90% strength to detect a 0.6 difference between an alpha coefficient for the nil hypothesis and at least of 0.7 for the alternative hypothesis, using a 2-tail hypothesis and a 5% significance level [25, 28].

For the assessment of test-retest reliability, means of the two (2) measurements were compared using the signed-rank test. In addition, Lin’s concordance correlation coefficient was estimated using the values of two (2) repeated measures from each subject. A 54-subject sample allows detecting a difference between coefficients 0.7 (nil hypothesis) and 0.85 (alternative hypothesis) with a 5% significance level and 80% strength [26, 29].

Convergent validity was evaluated by calculating Spearman correlation coefficients. A 70-subject sample size is adequate, considering values of at least 0.8 with a 95% confidence interval and a ±10 precision around the estimator.

To assess sensitivity to change, scores corresponding to repeated measurements were compared by using paired-t tests and a 5% significance level for the 2-tail hypothesis. For sample size estimation, an at least 10-point pre- and post- intervention difference with a 20-point standard deviation, a 5% significance level, and 80% strength were assumed; with such assumptions, 40 subjects were required.

Through Rasch analysis, the following aspects were evaluated [27, 28, 30, 31]: reliability indexes for persons and items (values ranging between 0 and 1); separation indexes (values ≥ 2 indicate proper separation); item-fit statistics (infit and outfit statistics). Items with infit-outfit > 1.4 and corresponding ZSD values > 2 are considered improperly fit; infit-outfit < 0.6 suggest item redundancy. For the rating scale diagnosis, means, outfit-infit mean squares, and step measures were estimated. Persons-items map distribution was also assessed. For the sample size in Rasch analyses, the recommendation of having at least 250 subjects when using Likert-type scales was followed [29, 32].

Confirmatory factorial analyses were done with the Stata® program; remaining statistical analyses were performed by means of the R program. The trial was carried out according to ethical considerations from the Helsinki Declaration and was approved by an institutional ethics committee. All of the patients gave their informed consent for participating in the trial, in a verbal form.

## Results

### Exploratory factorial analysis

Two-hundred and eight (56.2%) of 370 surveyed patients were males. Mean age was 57.9 years (SD 16.5). All of the patients were in a hemodialysis program; in the sample were included patients treated at facilities in all regions of the country: 236 from the central region (63.8%), 55 from the southwest (14.9%), 44 from the northwest (11.9%), 23 from the Caribbean coast (6.2%), seven (7) from the southeast (1.9%) and five (5) from the northeast (1.4%). Patients had a median time spent in renal replacement therapy of 3.4 years (interquartile range = 5.1 years). The main causes of renal disease were diabetes (34.3%, *N* = 127), hypertension (23.8%, *N* = 88) and glomerulonephritis (11.1%, *N* = 41). In 13.2% of patients (*N* = 49) the cause of kidney disease was unknown. The percentage of patients with Karnofsky scale <50 was 28.8% (*N* = 77). The median Charlson score was 6 (interquartile range = 6).

According to parallel analysis results, the optimal number of factors to analyze was 9.

Factorial structure showing best interpretability was that of orthogonal rotation Table 1.

**Table 1:** Factorial loads corresponding to Varimax rotation for ordinal variables

Item	D1	D2	D3	D4	D5	D6	D7	D8	D9	U2
Information offered at the renal unit about your rights and obligations	0.72									0.28
Flexible times and conditions according to your needs	0.71									0.33
Easiness for undergoing laboratory tests	0.67									0.42
Staff respect for your rights as a patient	0.64									0.39
Nice environment at the renal unit	0.61									0.45
Training offered for services provided at the renal unit	0.56									0.39
Compliance with date and time of scheduled appointments	0.56									0.53
Neatness and organization of the renal unit	0.54									0.58
Supplies quality and reliability	0.53									0.61
Compliance with connection/disconnection times	0.51									0.59
Reliability on the technology used for your therapy	0.49									0.53
Comfortable facilities at the renal unit	0.41									0.57
Reliability/credibility inspired by the nurse assistants		0.80								0.15
Nurse assistants’ behavior towards you		0.79								0.21
Nurse assistants being attentive to patients		0.77								0.25
Clarity of the information provided by nurse assistants about your treatment		0.74								0.19
Clarity of the information provided by the nurse about your treatment		0.55								0.38
Guidance provided about self-care and quality of life improvement			0.86							0.07
Reliability/credibility inspired by the psychologist			0.84							0.11
Warmth and kindness of the psychologist - listening to how you feel			0.71							0.26
Contact of the renal unit administrator with patients			0.37							0.62
Quality of the snack supplied at the renal unit			<0.3							0.75
Guidance and support offered for you to manage your social and family environment				0.74						0.22
Orientation and information about activities offered by the renal clinic				0.64						0.31
Social work personnel warmth and kindness				0.60						0.48
Timely response to any request or requirement				0.43						0.46
Easiness to communicate by phone with the renal unit				0.43						0.65
Kindness of the person who answers the phone at the renal unit				0.41						0.48
Reliability/credibility inspired by the physician/nephrologist					0.87					0.12
Physician/nephrologist behavior and bedside manners					0.79					0.25
Clarity of the information provided by the physician about your disease and treatment					0.68					0.35
Physicians are present to solve any issue					0.63					0.40
Assessment of your nutritional status						0.87				0.07
Warmth and kindness of the dietitian						0.77				0.24
Advice from the dietitian for you and your family to be able to apply						0.70				0.27
Timely drug delivery							0.74			0.36
Full medication delivery							0.72			0.38
Kindness of the pharmacist or person supplying the medications							0.71			0.32
Follow-up of your appointments by the admission assistant								0.77		0.14
Kindness of the admission assistant								0.71		0.22
Timing and clarity of guidance provided on requirements								0.56		0.31
Reliability/credibility inspired by nurses									0.68	0.17
Nurses are attentive to patients needs									0.58	0.32
Nurses’ behavior towards you									0.53	0.29

D1-D9: Analyzed domains. U2: Uniqueness

As it may be seen, one of the items (“Quality of the snack supplied at the renal unit”) has no adequate load values in any of the domains. Variance ratio for each factor was as follows: Domain 1: 0.22; Domain 2: 0.13; Domain 3: 0.11; Domain 4: 0.10; Domain 5: 0.09; Domain 6: 0.09; Domain 7: 0.09; Domain 8: 0.08; Domain 9: 0.07. Total variance resulting from the 9 Domains is 99%.

The nine (9) interpreted domains were as follows: Domain 1: Facilities and organization of the service. Domain 2: Care provided by attending nurses and/or assistants. Domain 3: Attention to psychological and administrative issues. Domain 4: Contact and social work personnel. Domain 5: Medical attention and care. Domain 6: Nutrition attention and care. Domain 7: Medications supply and quality. Domain 8: Features of the admissions process. Domain 9: Attention and care provided by head nurses.

### Confirmatory factorial analysis

Goodness-of-fit indicators were calculated for two (2) models: one corresponding to the first-order factorial structure presented in Table 1 and another following removal of the item “Quality of the snack supplied at the renal unit” and incorporation of covariances between some of the items, according to modification indexes outcomes; such indicators are presented in Table 2.

**Table 2:** Goodness-of-fit indicators from the confirmatory factorial analysis

	${\chi}^2/ d f$	RMSEA	CFI	TLI	AIC	BIC
Model 1^a^	2.8	0.070	0.876	0.864	24683.026	253217.013
Model 2^b^	2.4	0.065	0.897	0.890	23277.898	23915.799

^a^Model corresponding to the exploratory factorial analysis structure

^b^Model without Item P3.12.3 including correlations pointed out by modification indexes

**Fig. 1:**
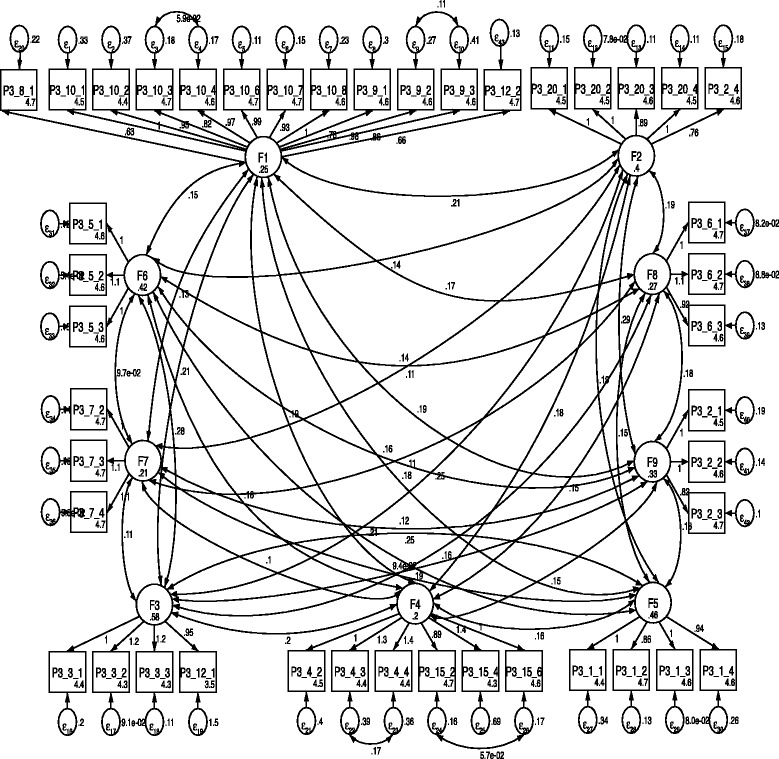
Model 2 structure

Despite these indicators, outcomes were similar for both models, thus suggesting an acceptable structure fit with 9 domains, CFI and TLI values are closer to 0.9, and RMSEA values, as well as information criteria, are lower than in model 2. The model structure with the best fit (*i.e.*, model 2) is depicted in Fig. 1.

### Internal consistency

Value of alpha coefficient for the whole scale was 0.96. There was no increase in such value following deletion of individual items. Alpha coefficient values for each of the domains were: Domain 1: 0.91 (12 items). Domain 2: 0.93 (5 items). Domain 3: 0.84 (4 items). Domain 4: 0.84 (6 items). Domain 5: 0.89 (4 items). Domain 6: 0.93 (3 items). Domain 7: 0.83 (3 items). Domain 8: 0.88 (3 items). Domain 9: 0.86 (3 items).

### Validity of convergent criteria

Results for correlation coefficients between the two applied scales (SDIALOR and ESUR-HD) are shown in Table 3, where it may be seen that correlations among the two scales’ domains reach low values (maximal being 0.33). However, all theoretical correlations have a plus sign and one of the highest values corresponds to the domain pair regarding medical care (*r* = 0.33). The domain with the largest number of correlations significantly different from 0 is the one regarding the admission process (correlated with domains 1, 3, 5, 6, and 7).

**Table 3:** Correlation coefficients among SDIALOR and ESUR-HD scales domains^a^

		ESUR								
		D1	D2	D3	D4	D5	D6	D7	D8	D9
SDIALOR ^b^	D1	0.23	0.18	0.20	0.18	0.33	0.22	0.12	0.27	0.23
	D2	0.09	0.18	0.09	0.03	0.20	−0.08	0.11	0.06	0.21
	D3	0.16	0.18	0.12	0.21	0.15	0.26	0.10	0.30	0.18
	D4	0.12	0.18	0.13	0.21	0.07	0.05	0.19	0.19	0.20
	D5	0.24	0.20	0.20	0.24	0.26	0.16	0.12	0.30	0.26
	D6	0.29	0.25	0.13	0.28	0.27	0.16	0.23	0.33	0.29
	D7	0.14	0.24	0.03	0.22	0.22	0.16	0.23	0.24	0.20

^a^Values corresponding to coefficients significantly different form 0 appear underlined

^b^Domain of the SDIALOR scale: D1: Medical care. D2: Nephrologist-GP interaction. D3: Facility and environment. D4: Accessibility. D5: Paramedic care. D6: Information provided by the physician. D7: Problem management

### Test-retest reliability

Mean time elapsed between the two (2) measurements in 54 patients was two days. Means obtained initially were similar to those obtained in the second measurement Table 4. There was no significant difference in any mean pair (signed-rank test, *p* > 0.05). Values for the concordance correlation coefficient for the scale were 0.85. When evaluating reliability within different instrument domains, low values were found for two (2) of them Table 4: domain 5 (medical personnel) and domain 7 (supplied medications).

**Table 4:** Results from repeated measurements and correlations coefficients for test-retest reliability assessment

Variable^a^	Measurement 1		Measurement 2		Correlation coefficient	
	p50	iqr	p50	iqr	Lin’s Rho	95% CI
Total Score	205.39	32.76	208.00	31.25	0.85	0.77–0.92
D1 Score	58.00	8.00	59.00	8.00	0.78	0.68–0.88
D2 Score	25.00	3.00	25.00	4.00	0.78	0.67–0.88
D3 Score	19.00	4.50	17.17	5.00	0.69	0.54–0.83
D4 Score	28.00	6.00	29.00	5.00	0.81	0.72–0.90
D5 Score	20.00	2.00	20.00	2.00	0.42	0.22–0.62
D6 Score	15.00	2.00	15.00	2.00	0.76	0.65–0.87
D7 Score	15.00	0.00	15.00	1.00	0.52	0.35–0.71
D8 Score	15.00	1.00	15.00	1.00	0.71	0.58–0.85
D9 Score	15.00	2.00	15.00	2.00	0.71	0.58–0.85

^a^Represents domains D1 to D9

### Sensitivity to change

Mean scores before and after the intervention (change of renal unit) corresponding to every scale domain, are presented in Table 5. For the 40 patients experiencing such intervention, differences turned out significant in the total scale scores as well as in the following domains: Facilities and organization of the service, Contact and social work personnel, and Medical attention and care (*p* < 0.05).

**Table 5:** Scores before and after a change in the renal unit

	Pre intervention		Post intervention	
	Mean	SD	Mean	SD
Total^*^	198.24	19.61	204.75	13.77
D1^*^	54.46	6.77	57.84	2.97
D2	23.34	2.80	23.80	2.26
D3	15.98	4.50	16.84	3.90
D4^*^	26.86	3.98	28.80	2.15
D5^*^	18.90	2.09	19.60	1.12
D6	14.58	1.28	14.30	1.43
D7	14.10	1.52	14.25	1.43
D8	14.36	1.37	14.53	1.09
D9	14.43	1.36	14.48	1.11

^*^Significant differences between pre and post median values (*p* < 0.05)

### Rasch analysis

Information about overall model fit is shown in Table 6. SD values from ZSTD > 2 suggest the presence of improperly fit items.

**Table 6:** Overall model fit indicators

		Infit		Outfit		Separation	Reliability
		MNSQ	ZSTD	MNSQ	ZSTD		
Persons	Median	1.24	0.3	1.09	0.0	3.18	0.91
	SD	0.70	1.9	0.75	1.9		
Items	Median	1.01	0.1	1.09	0.4	5.38	0.97
	SD	0.31	2.8	0.52	3.1		

Reliability indexes and those corresponding to persons and items separation for each of the domains are presented in Table 7. Reliability values > 0.57 and > 0.58 were found for items and persons, respectively. Modest separation indexes were found for persons but indexes were proper for items, which suggest restricted amplitude of the attribute in this patients’ sample.

**Table 7:** Persons and items separation indexes for the nine domains

		Reliability index	Separation index
D1	Person	0.74	1.68
	Item	0.92	3.40
D2	Person	0.88	2.11
	Item	0.90	2.96
D3	Person	0.69	1.50
	Item	0.99	8.78
D4	Person	0.58	1.18
	Item	0.96	5.21
D5	Person	0.71	1.57
	Item	0.96	4.62
D6	Person	0.70	1.54
	Item	0.68	1.46
D7	Person	0.58	1.18
	Item	0.57	1.15
D8	Person	0.64	1.32
	Item	0.82	2.20
D9	Person	0.66	1.39
	Item	0.94	4.02

Table 8 shows the fit statistics by weighted (infit) information criterion and extreme values (outfit) criterion for the items of the scale; it may be seen that four (4) of the items show an improper fit (“Snack”, “Contact with the administrator”, “Full medication delivery”, and “Easiness for phone communication”).

**Table 8:** Fit statistics for the scale items

	Items fit statistics			
	INFIT		OUTFIT	
Item	MNSQ	ZSTD	MNSQ	ZSTD
Physicians are present to solve any issue	1.11	1.10	1.44	3.10
Physician/nephrologist behavior and bedside manners	1.17	1.40	1.37	2.00
Reliability/credibility inspired by the physician/nephrologist	1.21	1.70	1.38	2.20
Clarity of the information provided by the physician about your disease and treatment	1.26	2.20	1.38	2.30
Nurses are attentive to patients needs	0.85	−1.40	0.95	−0.30
Reliability/credibility inspired by nurses	0.81	−1.80	0.84	−1.10
Nurses’ behavior towards you	0.74	−2.30	0.72	−1.70
Clarity of the information provided by the nurse about your treatment	0.77	−2.10	0.62	−2.60
Warmth and kindness of the psychologist - listening to how you feel	0.95	−0.50	0.83	−1.30
Guidance provided about self-care and quality of life improvement	1.04	0.40	0.95	−0.40
Reliability/credibility inspired by the psychologist	1.11	1.10	0.92	−0.60
Social work personnel warmth and kindness	1.23	2.00	1.31	2.00
Orientation and information about activities offered by the renal clinic	0.92	−0.80	1.17	1.30
Guidance and support offered for you to manage your social and family environment	0.91	−0.90	0.89	−0.80
Warmth and kindness of the dietitian	1.21	1.80	0.96	−0.20
Assessment of your nutritional status	1.13	1.20	0.94	−0.30
Advice from the dietitian for you and your family to be able to apply	1.04	0.40	0.86	−0.90
Kindness of the admission assistant	0.97	−0.20	0.68	−1.80
Follow-up of your appointments by the admission assistant	0.94	−0.50	0.68	−2.00
Timing and clarity of guidance provided on requirements	0.73	−2.50	0.61	−2.60
Timely drug delivery	1.28	2.10	1.89	3.70
Full medication delivery	1.55	3.80	2.59	6.10
Kindness of the pharmacist or person supplying the medications	0.92	−0.60	1.23	1.30
Supplies quality and reliability	1.16	1.20	1.28	1.30
Neatness and organization of the renal unit	1.03	0.30	1.03	0.30
Nice environment at the renal unit	1.01	0.10	1.04	0.30
Comfortable facilities at the renal unit	1.10	0.90	1.36	2.20
Compliance with date and time of scheduled appointments	0.94	−0.60	1.16	1.10
Compliance with connection/disconnection times	0.97	−0.30	1.44	3.00
Easiness for undergoing laboratory tests	0.90	−0.80	0.75	−1.40
Flexible times and conditions according to your needs	0.71	−2.80	0.66	−2.40
Information offered at the renal unit about your rights and obligations	0.68	−3.00	0.48	−3.70
Staff respect for your rights as a patient	0.78	−1.90	0.68	−2.00
Training offered for services provided at the renal unit	0.82	−1.60	0.69	−2.10
Contact of the renal unit administrator with patients	1.99	9.90	2.42	9.90
Reliability on the technology used for your therapy	0.65	−3.10	0.61	−2.30
Quality of the snack supplied at the renal unit	2.08	9.90	2.64	9.90
Kindness of the person who answers the phone at the renal unit	0.79	−1.80	1.13	0.70
Easiness to communicate by phone with the renal unit	1.45	4.20	1.82	5.70
Timely response to any request or requirement	0.68	−3.10	0.60	−2.90
Nurse assistants being attentive to patients	0.85	−1.40	1.12	0.90
Reliability/credibility inspired by the nurse assistants	0.70	−3.00	0.59	−3.20
Nurse assistants’ behavior towards you	0.83	−1.40	0.64	−2.40
Clarity of the information provided by nurse assistants about your treatment	0.70	−3.00	0.54	−3.70

Average scores Table 9, which are a mean value for the differences between the item’s ability and difficulty values, show an ascending monotonic trend in each of the domains, except for domains 1 and 9. This suggests that, except for those two domains, patients with the highest levels of satisfaction tend to grant the highest ratings to each item. This is consistent with the finding of fit values by weighted information criterion (infit) and extreme values criterion (outfit) out of the 0.6–1.4 range in the initial categories of such domains items. The presence of fit values that are not close to 1, especially for domains 1 and 9, suggests that people with high levels of satisfaction unexpectedly tend to give low ratings to such domains.

**Table 9:** Statistics for the scale categories

Domain category		Mean value	Infit MNSQ	Outfit MNSQ
D1	1	0.92	2.19	3.88
	2	0.28*	1.13	1.76
	3	0.96	1.01	1.2
	4	1.94	0.86	0.82
	5	3.51	0.92	0.94
	1	−4.45	0.42	0.43
	2	−1.18	1.33	1.41
D2	3	0.86	0.76	0.73
	4	3.74	0.99	1.03
	5	5.83	1.11	1.05
D3	1	−1.07	1.09	1.07
	2	−0.28	1.08	1.07
	3	0.33	0.86	0.78
	4	1.14	1.07	0.95
	5	2.07	1.06	1.15
D4	1	−0.22	1.38	1.87
	2	0.02	1.12	1.39
	3	0.44	0.9	0.93
	4	1.51	0.81	0.77
	5	2.8	1.02	0.99
D5	1	−2.1	1.31	1.38
	2	−1.22	0.87	0.84
	3	0.51	1.12	1.09
	4	2.62	0.95	0.82
	5	4.62	1.06	0.99
D6	1			
	2	−2.57	1.03	0.98
	3	−1.34	0.78	0.64
	4	2.66	0.93	0.95
	5	4.37	1.15	0.95
D7	1	−1.55	1.81	1.99
	2	−1.21	0.98	1.38
	3	0.61	1.03	0.99
	4	2.44	0.83	0.82
	5	4.02	0.97	0.92
D8	1	−2.27	1.1	1.01
	2	−1.73	0.9	0.77
	3	0.21	1.04	0.94
	4	3.51	0.83	0.81
	5	6.19	1.26	0.97
D9	1	0.53	2.31	3.93
	2	−2.06*	0.38	0.29
	3	0.06	0.8	0.69
	4	3.33	0.85	0.88
	5	5.54	1.12	1.02

*Without ascending monotonic trend

**Fig. 2:**
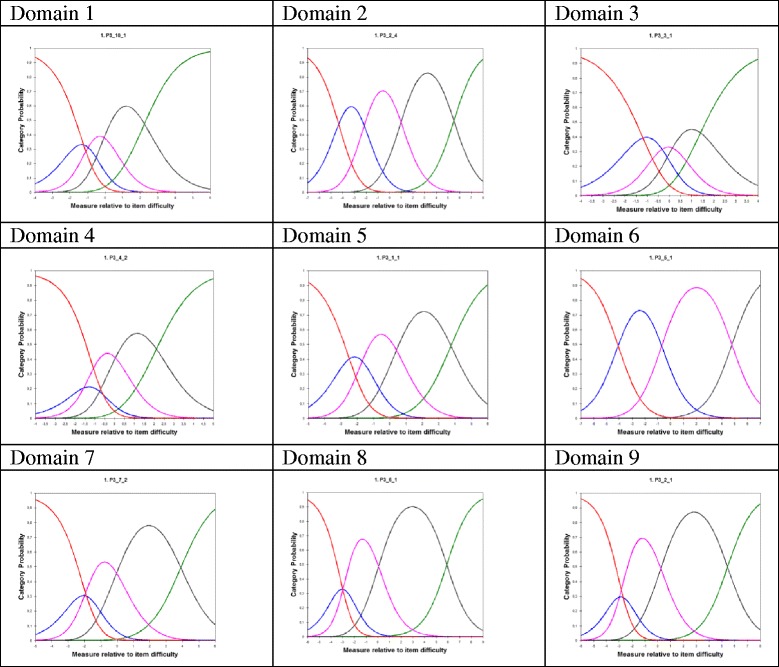
Categories probability curves

Probability curves for each item measuring category are shown in Fig. 2, grouped by domains; it may be seen that in 5 domains from category 2 (corresponding to the “unsatisfied” category in the Likert Scale) provides no clear discrimination of the underlying feature and might be disregarded.

**Fig. 3:**
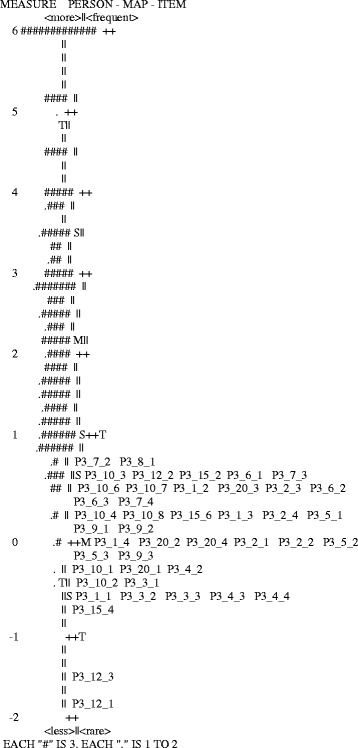
Persons-items map

The higher a patient is in the vertical scale Fig. 3, the higher is the degree of satisfaction. It may be seen that there is a group of patients with high levels of the attribute as well as an important dispersion in the measurements, especially for patients (range:−0.5 to 6 logits). It is also seen that means for items and persons (patients) are about 2 logits away, indicating that the latent feature presented by this group exceeds what may be measured by the scale (the map also reveals a ceiling effect). In addition, there are a couple of items (P3.12.1 and P3.12.3), which do not appear to adequately measure the attribute measured by other items. Other elements highlighted in the map are the strong marker items for the feature (P3_7_2 and P3_8_ in the upper part of the map) and the weak markers (P3_15_4). Distance between items P3_12_1 and P3_12_3 is consistent with their poor fit indicators.

## Discussion

Satisfaction with a dialysis service is a multidimensional attribute that in the ESUR-HD scale appears as a 9-factor or domains structure, adequately reflecting the underlying construct. Instruments designed for other clinical settings or different cultural environments focus on certain aspects or include elements that may not be applied in every culture. For example, the SDIALOR questionnaire assesses the interaction between the general practitioner and the nephrologist, an element that does not apply in many dialysis services in Colombia. On the other hand, such a questionnaire encompasses in just one domain what is related with the involvement of other healthcare professionals (dietician, social worker and psychologist), while in ESUR-HD, five (5) domains refer to this issue. Essur-HD is an instrument that can be employed by telephone, has a similar number of items than other instruments measuring the same construct, takes little time and can qualify in a simple way (only make summations of items without having to resort to complex transformations or algorithms for qualification).

Findings resulting from analyses undertaken to evaluate content validity suggest that the multidimensional structure named as “satisfaction” must be measured using instruments that are adequate for cultural particularities and specific setting services. This would render questionable the universal use of an instrument for measuring satisfaction with dialysis services in different countries.

Despite the fact that the ESUR-HD scale showed proper internal consistency, which suggests an adequate instrument reliability, such a finding should be taken with caution as the Cronback alpha coefficient tends to increase along with the number of items of an instrument (44 items in ESUR-HD). Consequently, this finding is to be analyzed considering other reliability indicators, such as those resulting from the theory response approach discussed later.

Regarding convergent validity (measured through the simultaneous application of two (2) instruments aimed at measuring the same construct), it was found that some scores for the scale domains are positively correlated with scores from other instrument domains measuring the same construct (*i.e.*, SLADIOR). This could favor the fact that the instrument has adequate concurrent validity; however, overall correlation values were low (the highest one being 0.33), and no correlation between apparently equivalent domains was found. For example, despite there being a positive, significantly different from 0 correlation between the “Medical care” domain in SLADIOR and “Medical attention” in ESUR-HD (*r* = 0.33), for domains “Facility and environment” in SLADIOR and “Facilities and service organization” in ESUR-HD, the correlation was 0.16 (which is not significantly different from 0). There was also a positive correlation different from 0 between the “Paramedic care” domain in SDIALOR and “Head nurse attention” in ESUR-HD (*r* = 0.26), but no significantly different from 0 correlation between “Paramedical care” in SDIALOR and “Nursing assistants care” or “Nutrition care” IN ESUR-HD was found. These findings correspond to low convergent validity and could simply reflect different latent variables structures in the two questionnaires or either that the two instruments are measuring the attribute from perspectives which are not precisely coincident (it must be borne in mind that SDIALOR is a more general instrument, as it also includes issues related to peritoneal dialysis). The described findings would favor the fact of satisfaction being a construct strongly influenced by cultural and local service particularities; another possible explanation for this finding is that the instrument SDIALOR not possess adequate psychometric properties when used in Colombia. Although it is the only instrument that has been cross-culturally adapted to measure satisfaction in renal patients in this country, this does not guarantee that it has proper validity and reliability for measuring a complex attribute, as with satisfaction; this means that in future studies on the psychometric properties of Essur-HD we should consider to evaluate the validity of the instrument using other scales with recognized measuring qualities [33].

Stability of scale scores in repeated measurements and the finding of a 0.85 concordance correlation coefficient under construct stability, also favor a proper reliability on the assessed instrument. These findings suggest that the overall variation of the instrument is mainly explained by the real variability of the construct being evaluated (patients’ satisfaction), and not so by error. Anyway, it is possible that the time between the two measurements (2 days) has been too short, and that the patients rather to respond an item de novo, had placed the value remembered of the first application. Low correlation values of the domains regarding medical care and supply and quality of medications may suggest these are less stable elements in the process of attention of hemodialysis patients.

The design used for evaluating the instrument’s ability to detect changes showed that scale scores were increased in most domains. This finding is consistent with the intervention performed: offering a group of patients the service in an enhanced facility with personnel changes, which implied a better service. Findings of differential changes depending on the domain (there were significant differences in total scores and in scores regarding facilities and service organization as well as with medical care, contact and social work personnel), suggest instrument scoring must be done considering the latent variables structure, as this strategy detects more specific change levels.

According to findings related with the Theory of item response (Rasch model), the sample of patients used for validating the scale rendered very high levels to the feature (there is a 2-logit difference between the means for item difficulty and patients ability; a ceiling effect of the measurement may be argued). This is consistent with the finding of low persons separation indexes as compared to items separation indexes. Rasch analysis findings also suggest that despite the fact the instrument may appropriately measure attribute levels in patients with lower degrees of satisfaction, in patients with attribute levels as high as those found in the sample, it might not discriminate adequately different attribute grades. In order for the instrument to have this property, including additional items would be required; doing so would demand a qualitative approach by including patients and other people associated with healthcare services. Previous studies have also had difficulties regarding a ceiling effect when measuring satisfaction among this kind of patients [20, 34]. In such cases, strategies such as increasing each item’s answer options and score normalization have been used [30]; however, using qualitative approaches to evaluate these constructs in patients reporting optimal experiences has also been proposed [20]. As a result of the findings from Rasch analysis in our study, we consider the most appropriate approach for improving the instrument might be incorporating other items that cover in a more convenient way the sample of renal replacement therapy user patients. Another finding from Rasch analysis is associated with the improper fit items: worst fit statistics were those regarding items “Quality of the snack offered at the renal unit” and “Contact with the unit administrator”. Despite the item regarding quality of the snack provided to patients results relevant in other instruments (SEQUS, SDIAOR), according to results from both classical measurement theory analysis and Rasch analysis, it has no adequate psychometric properties and should be excluded from the instrument being validated. Cultural factors are likely involved in Colombian patients not precisely associating a supplied snack with the quality of health service being provided. On the other hand, among hemodialysis services in Colombia, the administrative staff having direct contact with the healthcare personnel - but not with the patients - is commonplace. Thus, inquiring about the unit administrator as a marker of satisfaction with the service supplied may seem irrelevant to the patient. Although other improper fit items were detected, removing them from the instrument was not considered since the classical measurement theory approach did not diagnose them as problematic (resourcing to Rasch analysis results for removing an item from an instrument has not been recommended) [31, 35]. The scale has no redundant items (items with a proper fit, measuring the same attribute in a similar way). Items best representing the underlying dimensions (*i.e.*, obtaining higher scores probably reflect high levels of satisfaction) are “Timely medications delivery date” and “Supplies quality and reliability.” On the other hand, the item evaluating the “Easiness for phone communication with the renal clinic” is a weak marker of the attribute (even low satisfied patients may grant it a high score). Regarding measurement scale of the items, it was found that it discriminates adequately among different intensity levels of the attribute, but may be restricted by suppressing the “unsatisfied” option, as in several domains this category does not properly discriminate the attribute intensity.

We note the following limitations of our study:

1. The ceiling effect has an impact on the ability of the instrument to differentiate patients with high levels of the attribute. This can be problematic as far as assessing sensitivity to change, given the weight that this scenario would have on the phenomenon of regression to the mean.

2. The time taken for evaluating the reliability test-retest may have favored the finding of high levels of correlation, which may not necessarily reflect the reliability of the construct.

3. Concurrent validity could be affected by the use of an instrument whose psychometric properties are not clearly known in Colombia.

## Conclusion

According to the results of the present study, the ESUR-HD scale measures patients’ satisfaction with hemodialysis therapy as if it was a 9-domain construct. The 44–item version includes a measuring scale that must be adjusted by removing the “unsatisfied” category and deleting 2 items showing an improper fit (“Quality of the snack offered at the renal unit” and “Contact with the unit administrator”). The instrument showed acceptable validity and reliability; in addition, it was able to detect in the construct changes following an intervention that improved the patients’ satisfaction. Using an items measurement scale with just 4 categories allows adequate detection of different attribute levels. Including new items allowing improved discrimination between high satisfaction levels in subsequent scale versions is recommended.

## Abbreviations

ESUR-HD: Scale for Evaluation of Hemodialysis Patient’s Satisfaction with Service provided at a Chronic Kidney Disease Unit
SDIALOR: Satisfaction des patients dialysés en Lorraine
TLI: Tucker-Lewis index
CFI: Comparative fit index
RMSEA: Root mean square error of approximation
BIC: Bayesian information criteria
AIC: Akaike information criteria

## References

[1] Nguyen Thi PL, Frimat L, Loos-Ayav C, Kessler M, Briancon S (2008). SDIALOR: a dialysis patient satisfaction questionnaire. Nephrol Ther.

[2] Donabedian A (1988). The quality of care. How can it be assessed?. JAMA.

[3] Gasquet I (1999). Patient satisfaction and hospital performance. Presse Med.

[4] Carr-Hill RA (1992). The measurement of patient satisfaction. J Public Health Med.

[5] Bodenheimer T (1999). The American health care system--the movement for improved quality in health care. N Engl J Med.

[6] Chassin MR (1993). Improving quality of care with practice guidelines. Front Health Serv Manage.

[7] Chassin MR (1996). Quality of health care. Part 3: improving the quality of care. N Engl J Med.

[8] Chassin MR (1996). Improving the quality of health care: what strategy works?. Bull N Y Acad Med.

[9] Wasserfallen JB, Halabi G, Saudan P, Perneger T, Feldman HI, Martin PY (2004). Quality of life on chronic dialysis: comparison between haemodialysis and peritoneal dialysis. Nephrol Dial Transplant.

[10] Kirchgessner J, Perera-Chang M, Klinkner G, Soley I, Marcelli D, Arkossy O (2006). Satisfaction with care in peritoneal dialysis patients. Kidney Int.

[11] Kimmel PL (2000). Psychosocial factors in adult end-stage renal disease patients treated with hemodialysis: correlates and outcomes. American J Kidney Dis.

[12] Kimmel PL (2001). Psychosocial factors in dialysis patients. Kidney Int.

[13] Kimmel PL (2005). Psychosocial factors in chronic kidney disease patients. Semin Dial.

[14] Juergensen PH, Zemchenkov A, Watnick S, Finkelstein S, Wuerth D, Finkelstein FO (2002). Comparison of quality-of-life assessment in Russia and the United States in chronic peritoneal dialysis patients. Advances in peritoneal dialysis Conference on Peritoneal Dialysis.

[15] Kovac JA, Patel SS, Peterson RA, Kimmel PL (2002). Patient satisfaction with care and behavioral compliance in end-stage renal disease patients treated with hemodialysis. Am J Kidney Dis.

[16] Wuerth DB, Finkelstein SH, Kliger AS, Finkelstein FO (2000). Patient assessment of quality of care in a chronic peritoneal dialysis facility. Am J Kidney Dis.

[17] Rubin HR, Jenckes M, Fink NE, Meyer K, Wu AW, Bass EB (1997). Patient’s view of dialysis care: development of a taxonomy and rating of importance of different aspects of care. CHOICE study. Choices for Healthy Outcomes in Caring for ESRD. Am J Kidney.

[18] Wasserfallen JB, Moinat M, Halabi G, Saudan P, Perneger T, Feldman HI (2006). Satisfaction of patients on chronic haemodialysis and peritoneal dialysis. Swiss Med Wkly.

[19] Rubin HR, Fink NE, Plantinga LC, Sadler JH, Kliger AS, Powe NR (2004). Patient ratings of dialysis care with peritoneal dialysis vs hemodialysis. JAMA.

[20] Van der Veer SN, Jager KJ, Visserman E, Beekman RJ, Boeschoten EW, de Keizer NF (2012). Development and validation of the Consumer Quality index instrument to measure the experience and priority of chronic dialysis patients. Nephrol Dial, Transplant.

[21] Barendse SM, Speight J, Bradley C (2005). The Renal Treatment Satisfaction Questionnaire (RTSQ): a measure of satisfaction with treatment for chronic kidney failure. Am J Kidney Dis.

[22] Wood R, Paoli CJ, Hays RD, Taylor-Stokes G, Piercy J, Gitlin M (2014). Evaluation of the consumer assessment of healthcare providers and systems in-center hemodialysis survey. Clin J Am Soc Nephrol.

[23] Sanabria M, Tobón J, Certuche MC, Sánchez R (2015). Adaptación transcultural del cuestionario SDIALOR para su utilización en Colombia. Rev Fac Med Univ Nac Colomb.

[24] Streiner DL, Norman GR (2008). Health measurement scales : a practical guide to their development and use.

[25] Crawford AV, Green SB, Levy R, Lo WJ, Scott L, Svetina D (2012). Evaluation of Parallel Analysis Methods for Determining the Number of Factors. Educ Psychol Meas.

[26] Hu LT (1999). Cutoff criteria for fit indexes in covariance structure analysis: Conventional criteria versus new alternatives. Struct Equ Model.

[27] McCallum R, Browne M, Sugawara H (1996). Power Analysis and determination of samle size for covariance structure modeling. Psychol Methods.

[28] DBonett D (2002). Sample size requirements for testing and estimating coefficient alpha. J Educ Behav Stat.

[29] Lin LIK, Hedayat A, Wu W (2012). Statistical tools for measuring agreement.

[30] Linacre JM (2002). Optimizing rating scale category effectiveness. J Appl Meas.

[31] Wright B, Linacre M (1994). Reasonable mean-square fit values. Rasch Measurement Transactions.

[32] DeMars C (2010). Item response theory.

[33] Weidmer BA, Cleary PD, Keller S, Evensen C, Hurtado MP, Kosiak B (2014). Development and evaluation of the CAHPS (Consumer Assessment of Healthcare Providers and Systems) survey for in-center hemodialysis patients. Am J Kidney Dis.

[34] Moret L, Nguyen JM, Pillet N, Falissard B, Lombrail P, Gasquet I (2007). Improvement of psychometric properties of a scale measuring inpatient satisfaction with care: a better response rate and a reduction of the ceiling effect. BMC Health Serv Res.

[35] Karabatsos G (2000). A critique of Rasch residual fit statistics. J Appl Meas.

